# Region-specific interneuron demyelination and heightened anxiety-like behavior induced by adolescent binge alcohol treatment

**DOI:** 10.1186/s40478-019-0829-9

**Published:** 2019-11-08

**Authors:** James Rice, Laurence Coutellier, Jeffrey L. Weiner, Chen Gu

**Affiliations:** 1 Department of Biological Chemistry and Pharmacology, The Ohio State University, 182 Rightmire Hall, 1060 Carmack Road, Columbus, OH 43210 USA; 2 Department of Psychology, The Ohio State University, Columbus, OH 43210 USA; 3 Department of Physiology and Pharmacology, Wake Forest University, Winston-Salem, NC 27101 USA

**Keywords:** Adolescent binge ethanol treatment (ABET), Anxiety, Gray matter myelin, Hippocampus, Medial prefrontal cortex (mPFC), Parvalbumin-positive (PV+) GABAergic interneuron

## Abstract

Adolescent binge drinking represents a major public health challenge and can lead to persistent neurological and mental conditions, but the underlying pathogenic mechanisms remain poorly understood. Using a mouse model of adolescent binge ethanol treatment (ABET), we found that this treatment induced behavioral changes associated with demyelination in different brain regions. After ABET, adolescent mice exhibited anxiogenic behaviors with no change in locomotion on the elevated plus maze, and impaired spatial memory indicated by a significant reduction in spontaneous alternation in the Y maze test. Both effects persisted into adulthood. Anatomical studies further showed that ABET induced a significant reduction of parvalbumin-positive (PV+) GABAergic interneurons and myelin density in the hippocampus and medial prefrontal cortex (mPFC). While these deficits in PV+ interneurons and myelin persisted into early adulthood in the hippocampus, the myelin density recovered in the mPFC. Moreover, whereas ABET mainly damaged myelin of PV+ axons in the hippocampus, it primarily damaged myelin of PV-negative axons in the mPFC. Thus, our findings reveal that an adolescent binge alcohol treatment regimen disrupts spatial working memory, increases anxiety-like behaviors, and exerts unique temporal and spatial patterns of gray matter demyelination in the hippocampus and mPFC.

## Introduction

Binge drinking is defined by the National Institute on Alcohol Abuse and Alcoholism as “a pattern of drinking that brings blood alcohol concentration levels to 0.08 g/dL”. This typically occurs after consuming 4–5 standard drinks within a 2 h period. Among all age groups, adolescents are the most likely to binge drink [13, 20]. Meta-analysis showed that approximately 20–40% of adolescents engaged in binge drinking, and in particular, about 10% of 12th graders and 20% of college students who drink alcohol are heavy binge drinkers [28, 41]. Large population studies have found that, across the teenage years, both early age of first use and binge drinking predict increased risk of lifetime alcohol use disorder (AUD) and alcohol-related violence and injuries [20, 41]. Despite the strong association between adolescent binge drinking and AUD, the neural substrates underlying this relationship remain poorly understood.

Alcohol binge drinking can be especially harmful in adolescents, since adolescence is a critical developmental period associated with maturation of cognitive ability, personality, and frontal cortical executive functions. This coincides with gray matter (GM) myelination in different brain regions, including the hippocampus and medial prefrontal cortex (mPFC). GM myelination is a long process that continues into adulthood in both humans and rodents and its disruption can lead to various neurological disorders [1, 2, 30]. Different from white matter (WM) myelin that has been extensively studied, GM myelin often localizes adjacent to neuronal soma, dendrites and synapses. The hippocampus plays a key role in spatial memory and anxiety [43], and is known as one of the most sensitive targets for the neurotoxic effects of ethanol (EtOH) [46]. The mPFC is involved in planning and decision making, and reciprocally connected to the hippocampus and other regions that mediate positive and negative reinforcement [47, 51]. Alcohol-mediated alterations in mPFC connectivity may lead to loss of control over attention and emotion, and to increased engagement in risky behaviors, such as binge drinking [40, 48]. Chronic EtOH exposure has been shown to reduce myelin protein expression, leading to demyelination in WM that is commonly observed in human alcoholics [6, 25, 32, 36, 53].

Recent studies discovered that the locally-projecting, parvalbumin-positive (PV+) GABAergic interneurons contribute a major portion of myelinated axons within the cortex and hippocampus [29, 42]. These interneurons play a key role in maintaining proper excitatory/inhibitory balance and high-frequency network oscillations via feedback and feedforward inhibition [8, 15, 21]. PV+ interneurons can fire action potential up to 1 kHz and thus they are also called fast-spiking interneurons [19, 21]. They target excitatory neurons, and receive strong excitatory input, as well as inhibition from other PV+ interneurons [8]. PV+ interneuron reduction is associated with cognitive and emotional problems in mice and humans [7, 24, 26]. However, the possible impact of chronic EtOH on GM and interneuron myelin in regions like the hippocampus and mPFC remains unknown.

To address this critical gap in our knowledge, we established a mouse adolescent binge EtOH treatment (aka ABET) model with an on-off-on intermittent temporal pattern [9]. Prior studies revealed that this model led to long-lasting changes in adulthood, including disrupted Barnes Maze reversal learning, volume enlargements in specific brain regions (but no changes in total brain volume) including the orbitofrontal cortex, cerebellum, thalamus, internal capsule and genu of the corpus callosum, and increased expression of extracellular matrix proteins [9]. Other studies have also revealed persistent effects of adolescent alcohol exposure on brain function and structure including increased EtOH intake and risk-taking preference, impaired conditioned discrimination and object recognition [14, 34], and frontal cortical damage [11]. EtOH binge treatment also reduced myelin density in the mPFC in adolescent rats, suggesting that greater severity of PFC WM neuropathology may be correlated with higher levels of relapse-like drinking in adulthood [44]. Thus, ongoing myelination in the adolescent brain may make binge drinkers particularly vulnerable to long-term consequences of chronic alcohol exposure, including cognitive deficits and addiction. However, the mechanism and function of myelin alterations induced by adolescent EtOH binge exposure remain poorly understood.

Using behavioral testing and anatomical analysis, we have found that ABET induces long-lasting behavioral changes associated with differential demyelination in hippocampal and mPFC circuits. Thus, dysregulation of GM myelination in cortical and limbic regions may contribute to maladaptive behavioral phenotypes that develop following adolescent binge alcohol exposure.

## Materials and methods

### Animals

**Fig. 1:**
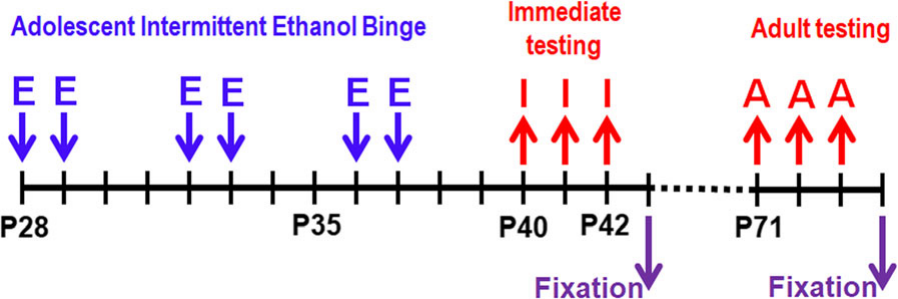
Adolescent binge ethanol model and testing schedul. Male mice were given either saline or EtOH (E) (5 g/kg, i.g. 25% ethanol w/v) once a day during adolescence (P28-P37) in an intermittent fashion. Immediate behavioral testing (I), behavioral testing in early adulthood (A), and fixation for anatomical studies were performed

Male adolescent C57Bl6 mice were obtained from the Jackson Laboratory (Bar Harbor, Maine, USA) and arrived at postnatal day 21 (P21). Mice were group housed (2–5 mice per cage) and maintained on a standard 12-h light-dark cycle with lights on at 6 am. Mice were given 1 week to acclimate to the animal facility before EtOH exposure began and had ad libitum access to food and water throughout the duration of the study. All animal handling procedures in the current study were approved by the Ohio State University Institutional Animal Care and Use Committee (IACUC) and conformed to the United States National Institutes of Health Guide for the Care and Use of Laboratory Animals. After acclimation, mice were randomly assigned by cage to receive gavage doses of saline or EtOH and then subjected to behavioral testing either 3 or 34 days after the final EtOH exposure as described in Fig. 1.

### EtOH binge treatment via Intragastric intubation

Mice received either saline or 25% (vol/vol) EtOH (5 g/Kg) via intragastric (i.g.) intubation on days P28, P29, P32, P33, P36, and P37. Previous studies utilizing this EtOH exposure paradigm reported blood EtOH concentrations (BECs) of ~ 300 mg/dL in C57Bl6 mice [9]. To confirm that this EtOH binge model results in a similar level of BECs in our lab, we used the EtOH Assay Kit (Cat. #: ab65343; Abcam, Cambridge, MA, USA) to measure BECs at 1.5 h post i.g. intubation (~ 280 ± 20 mg/dL). Thus, the BECs of this EtOH binge model in our lab are consistent with what was previously reported.

### Behavioral testing

A few days or a month after the final EtOH exposure, mice were subjected to behavioral testing in the following order: Elevated plus maze (EPM), Y-maze spontaneous alternation, and Open Field on consecutive days. All behavioral testing occurred during the light phase. Prior to behavioral testing, mice were acclimated to the procedure room for at least 1 h. Home cages were then moved to an adjacent room and mice were transported to the procedure room for testing one at a time. When testing was complete, mice were returned to their home cage and the apparatus was cleaned using a 70% EtOH solution between trials. Mouse behavior was recorded by a mounted video camera (Sony Corporation, Tokyo, Japan) and stored for offline analysis by hand or using Ethovision XT 12 software (Noldus Information Technology, Wageningen, The Netherlands).

#### Elevated plus maze

Anxiety-like behavior was measured using the EPM as described previously [33]. EPM testing was carried out on a raised (74 cm) plus-shaped maze with four arms (34 cm long). Two opposite arms were “open” with no walls; the other two arms were “closed” by opaque walls (22 cm high). Mice were placed in the center of the maze at the intersection of the two arms and allowed to explore the maze for 5 min. Total distance traveled, arm entries, and time spent on the four arms were recorded, with percentage time spent in the open arms/(open + closed arms) of the EPM used as a measure of anxiety-like behavior. Time spent in the center was not included in the analysis.

#### Y-maze spontaneous alternation

Working memory was assessed using the spontaneous alternation Y-maze test as described previously [10, 38]. Mice were placed at the end of one arm of a symmetric Y maze with arms measuring 40 cm long, 8 cm wide, and 20 cm high and allowed 5 min to freely explore the apparatus. The series of arm entries (e.g. ACBCABCBCA) was recorded using an overhead camera. An alteration is counted when the mouse entered the 3 different arms during a triad on overlapping triplet sets (e.g. in the sequence ACBCABCBCA, five alternations were recorded). Spontaneous alternation was measured by counting the number of times the mouse entered each of the 3 arms of the maze in succession divided by the maximum number of possible alternations, where maximum alternations was calculated as the total number of arm entries minus 2.

#### Open field

Locomotor activity was measured using the open field task [39]. Mice were placed in the center of a square arena (40 cm X 40 cm) and behavior was recorded for 10 min. Total distance traveled per 1 min time bins was used as a measure of locomotion, and time spent in the center (25 cm X 25 cm) and perimeter of the arena were also recorded.

### Brain tissue preparation

Twenty four hours after the completion of behavioral testing, all mice were deeply anesthetized with avertin (250 mg/kg) and perfused transcardially with 20 ml of PBS followed by 20 ml of a 4% formaldehyde/PBS solution. Brains were removed and post-fixed for 1 h in 4% formaldehyde/PBS solution and then cryoprotected in a 30% sucrose/PBS solution for > 24 h. Tissue blocks were made using optimal cutting temperature (OCT) medium (Braintree Scientific, Braintree, MA, USA) and samples were stored at − 80 °C until being cut using a Microm HM55 cryostat (Thermo Scientific, Waltham, MA, USA). Forty micrometer coronal sections were cut and mounted on Superfrost Plus slides (FisherScientific, Pittsburgh, PA, USA) and stored at − 20 °C.

### Antibodies and reagents

The following antibodies were used in the present study: rat monoclonal anti-myelin basic protein (MBP; Cat. #: MAB386; Millipore, Billerica, MA); rabbit anti-parvalbumin (PV; Cat. #: 195002; Synaptic Systems, Gottinggen, Germany); rabbit anti-degraded MBP (dMBP; Cat. #: AB5864; Millipore, Billerica, MA); Cy3-, and Cy5-conjugated secondary antibodies (Jackson ImmunoResearch Laboratories, West Grove, PA, USA). All antibodies were used in a 1:200 dilution. The nuclear dye Hoechst 33342 was purchased from Invitrogen (Cat. #: H3570; Carlsbad, CA, USA).

### Immunohistochemistry

Mouse brain sections were stained as detailed in previous reports from our laboratory [4, 5, 18, 22, 23]. Sections were permeabilized in 1% Triton X/PBS for 1 h at room temperature (RT) and then blocked with 2.5% normal donkey or goat serum for 1 h at RT. Sections were then incubated overnight at 4 °C with the primary antibodies in blocking buffer. Twenty-four h later, sections were rinsed for 5 min seven times, incubated with the appropriate secondary antibodies in blocking buffer for 3 h at RT, counterstained with Hoechst 33342 for 10 min, and again rinsed for 5 min seven times. Stained slides were coverslipped with tris-buffered Fluoro-Gel mounting media (Electron Microscopy Sciences, Hatfield, PA, USA). Multiple staining rounds were carried out with at least one slide from each experimental group.

### Microscopy and image quantification

Fluorescence microscopy and image analyses were carried out as described in previous publications from our laboratory [17, 18, 22, 23, 31, 49]. We focus on the following brain regions, dorsal hippocampus (Bregma between − 1.82 and − 2.30 mm) and mPFC (Bregma between + 1.98 and + 1.78 mm). Low magnification images were captured with a Spot CCD camera RT slider (Diagnostics Instruments, Sterling Heights, MI, USA) on a Zeiss Axiophot upright microscope using a 20X/0.50 Plan Apo objective and saved as 12-bit TIFF files. Exposure times were adjusted to ensure that pixel intensity in targeted tissue samples were below saturation, and exposure time was kept constant across all experimental conditions for each of the specific fluorophores utilized. Representative high magnification images were captured with a Leica TCS SL confocal imaging system (Leica Microsystems, Mannheim, Germany) using a 100X HCX Plan Apo CS oil immersion objective with a numerical aperture of 1.40, or on a Andor Revolution WD spinning disk confocal system (Oxford Instruments, Abingdon-on-Thames, UK) based on a Nikon TiE inverted microscope using a 60X CFI Plan Apo VC water immersion objective with a numerical aperture of 1.40. Z-stack images (8-bit TIFF files) were taken for each region of interest at ~ 0.25 μm steps and flat images were generated using a maximum intensity projection.

Image analyses were performed with NIH ImageJ (Fiji) software on low magnification images captured with 20X0.50 objective. The same staining procedures, imaging exposure times and brain regions were used across the sections from different conditions. PV+ cell counts were carried out by overlaying PV stained sections over the nuclear dye Hoechst stained sections. The number of nuclei that overlapped with PV expression were counted manually and used as a measure of PV+ cells (here, AU = cell number/image field) for subsequent analysis. Each image at 20x is 1200 × 1600 pixels, with a measure of 0.37 μm per pixel. Thus, 1 image field = 444 × 592 μm^2^ ~ 0.26 mm^2^. To quantify the density of MBP+/dMBP+ myelin segments and PV+ processes in an image field, each image was thresholded to reveal MBP+ myelin segments or PV+ neuronal processes, and then binarized as previously described [5, 31], for either density quantification (segments or processes/image field) or further quantification of colocalization. In order to determine the extent to which alterations in MBP+ myelin segment density might be related to changes in PV+ processes after ABET, correlations were calculated for overlapping pixels from PV and MBP images. A Pearson’s r was calculated for each set of images, and mean correlation values were compared across treatment conditions. For all dependent measures, at least 3 slides per region of interest per mouse were analyzed from ≥3 mice per experimental condition. The n numbers for analyzed slices are provided in figure legends. All image analysis and quantification have been verified and supported with confocal microscopy.

### Statistical analysis

Results are presented as mean ± SEM. Two-way ANOVAs with condition (Control vs. EtOH) and age (Adolescent vs. Adult) as between-subjects factors were carried out using SigmaPlot 13.0 software (Systat Software Inc., Chicago, IL, USA). Tukey’s post hoc tests were used to compare simple mean effects when following significant interaction effects. One-sample t-test was carried out in the Y maze test to compare each condition with the chance level (50%) as previously reported [10], and Pearson’s r was calculated for PV/MBP correlation analyses. The Type I error rate was set at 0.05.

## Results

### ABET induced anxiety-like behaviors that persisted into adulthood

To determine behavioral and brain structural changes associated with adolescent alcohol binge drinking, we used an established mouse model of ABET [9] with some modifications (Fig. 1). Male C57Bl6 mice received either saline or 25% (vol/vol) EtOH (5 g/kg) via intragastric (i.g.) intubation on days P28, P29, P32, P33, P36, and P37, mimicking human adolescent binge drinking in an on-off-on intermittent temporal pattern (Fig. 1). Right after EtOH intubation, mice transiently displayed moderate sedation and poor coordination, closely resembling some of the physical behavioral characteristics observed in humans after binge drinking. To control for any stress associated with the intubation procedure, control mice received the equivalent volumes of i.g. saline. All mice were subjected to three behavioral assays, including the EPM, Y maze and open-field test, either 3 (Adolescent) or 34 (Adult) days after the final treatment (Fig. 1). We used these three behavioral assays to assess potential acute and long-lasting effects of ABET on general locomotion, cognition and anxiety-like behavior in adolescent mice.

**Fig. 2:**
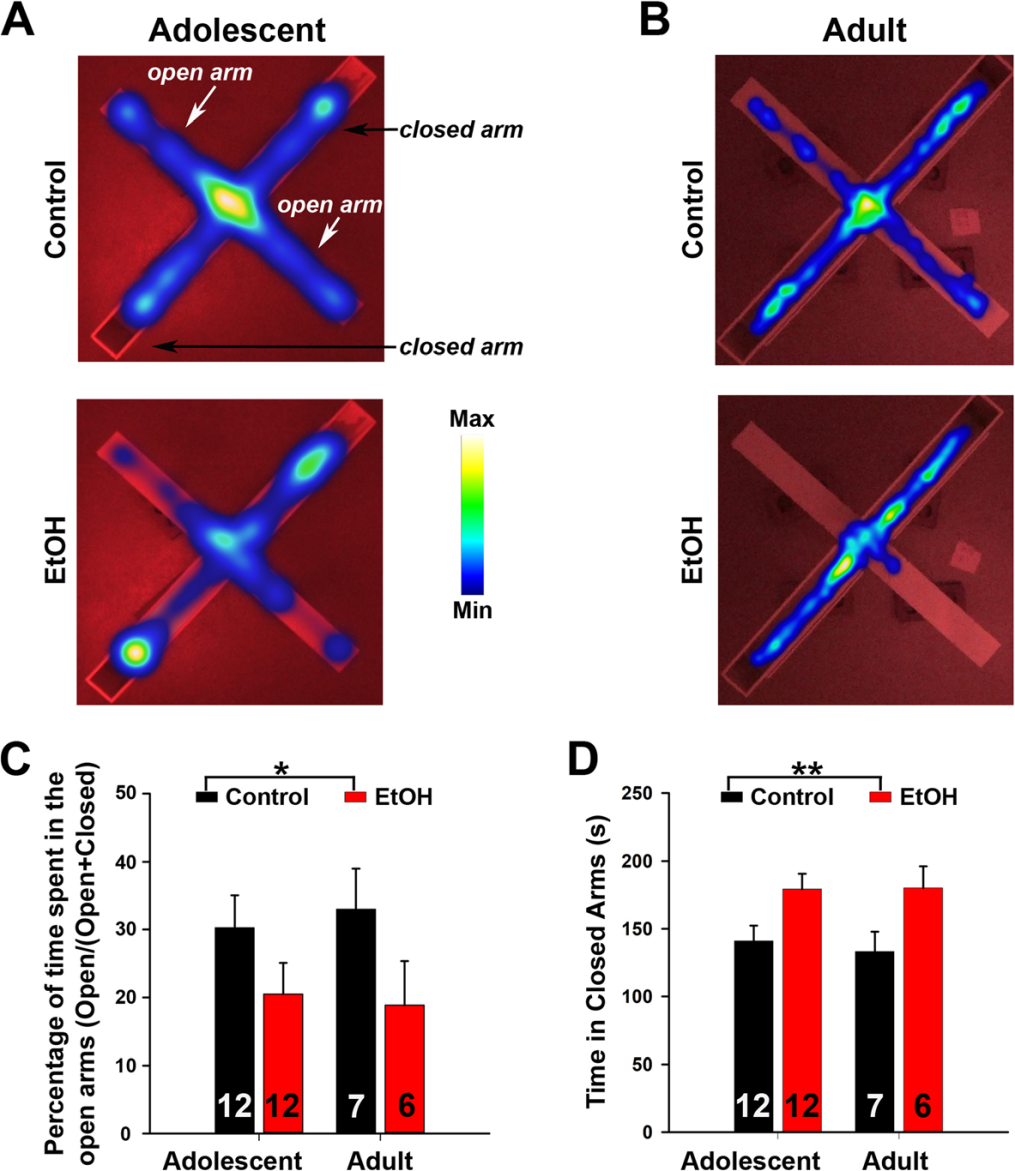
The EPM test revealed ABET-induced anxiety-like behavior. **a** The top view of time spent in the open and closed arms of the EPM immediately after (Adolescent) mice received saline (top) or EtOH (bottom). Time spent on the EPM is shown in heat maps. **b** EPM heat maps for the testing about a month after (Adult) mice received saline (top) or EtOH (bottom). **c** Summary for percentage time spent in the open arms/(open + closed arms) for both Adolescent and Adult testing. Two-way ANOVA revealed a significant effect of the condition for time spent on the open arms [F_1,33_ = 4.66, *p* = 0.039]. There was no effect of the age [F_1,33_ = 0.01, *p* = 0.92], nor the condition and age interactions [F_1,33_ = 0.15, *p* = 0.70]. *, *p* < 0.05. **d** Summary for time spent on the closed arms for both Adolescent and Adult testing. Two-way ANOVA revealed a significant effect of the EtOH treatment for time spent on the closed arms [F_1,33_ = 10.01, *p* = 0.003]. There was no effect of the age (Adolescent vs. Adult) [F_1,33_ = 0.07, *p* = 0.8], nor the condition (Control vs. EtOH) and age interactions [F_1,33_ = 0.11, *p* = 0.75]. **, *p* < 0.01. The n number is provided within each bar

Among the three behavioral tests, the EPM was performed first. The EPM is a well-validated test of unconditioned anxiety-like behavior with decreased open arm time and open arm entries typically reflecting an anxiogenic phenotype. The control adolescent mice spent significant amount of time in the open arms, but the time spent in the closed arms were still relatively more (Fig. 2a). ABET caused significant decrease of percentage time in the open arms/(open + closed arms) in adolescent mice and at the same time significant increase of time spent in the closed arms (Fig. 2a, c, d). After treated mice grew into early adulthood, these differences persisted (Fig. 2b-d). So both adolescent and adult mice with ABET spent significantly less percentage time in the open arms/(open + closed arms) [F_1,33_ = 4.66, *p* = 0.039, two-way ANOVA] and significantly more time in the closed arms [F_1,33_ = 10.01, *p* = 0.003, two-way ANOVA] in the EPM compared to controls (Fig. 2c,d). There was no significant difference caused by age (Adolescent vs. Adult) or the condition (Control vs. EtOH) x age interactions for percentage time in the open arms/(open + closed arms) and closed arm time (Fig. 2). There was no significant difference for total distance travels between the groups (unit in cm; Adolescent Control: 1286 ± 95; Adolescent EtOH: 1153 ± 63; Adult Control: 1209 ± 50; Adult EtOH: 1094 ± 79). Taken together, these results indicate that ABET significantly increases anxiety-like behavior, which lasts into early adulthood.

### ABET reduced spatial working memory and had no effect in locomotion

**Fig. 3:**
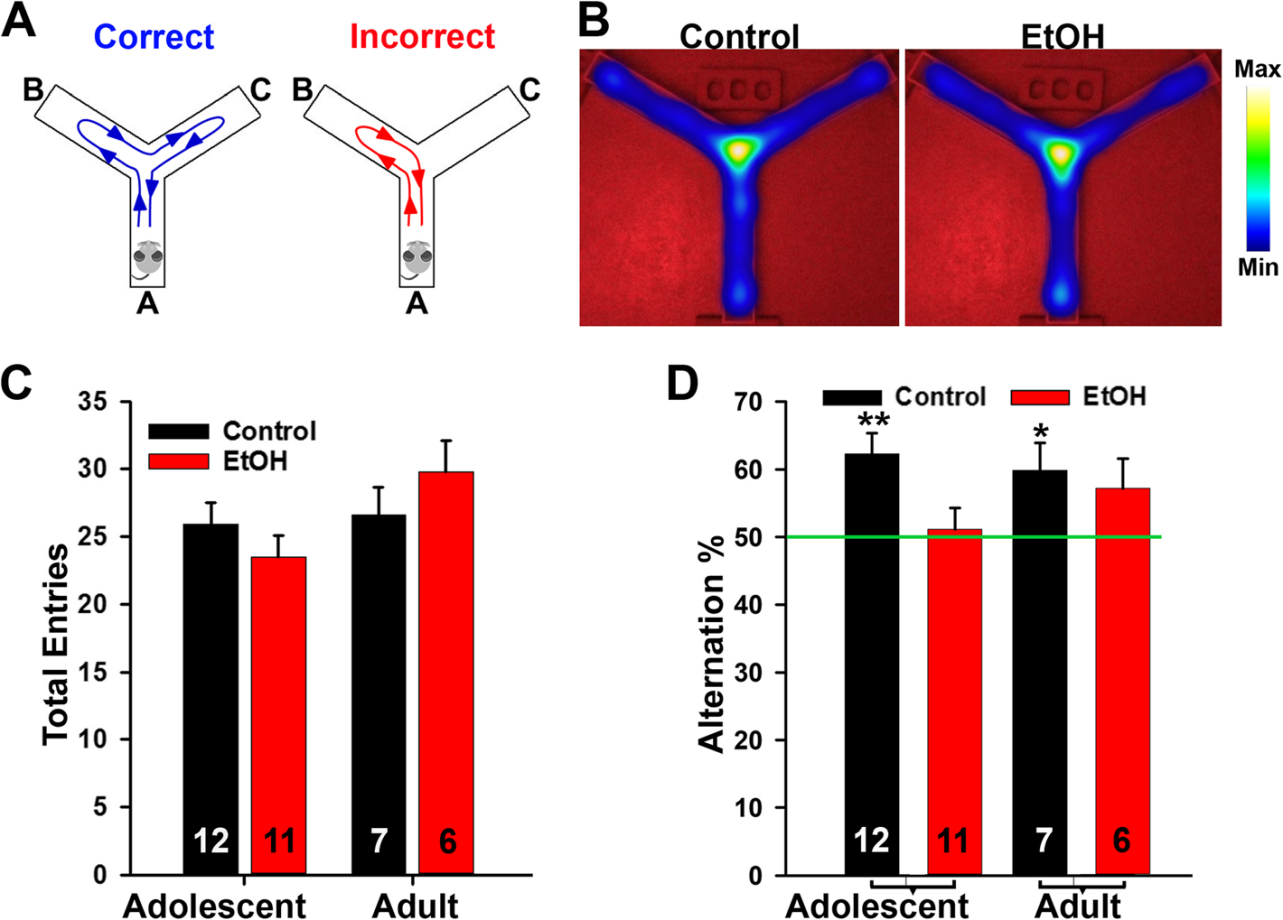
The Y maze test showed that spontaneous alternation was impaired by ABET. **a** Diagrams for the Correct (left) and Incorrect (right) alternation in the Y-maze test. **b** The top view of the Y maze and the overall activities shown by heat maps for a control mouse (left) and an ABET mouse (right). **c** Summary of the total arm entries. Two-way ANOVA for condition [F_1,32_ = 0.04, *p* = 0.84], age [F_1,32_ = 3.39, *p* = 0.08], and condition x age [F_1,32_ = 2.25, *p* = 0.14]. **d** Summary of spontaneous alternation. The percent of alternation in the Y-maze was significantly above chance level (50%) in Control but not EtOH mice in both adolescence and adulthood (One-sample t-test: Adolescent Control, *p* = 0.004; Adolescent EtOH, *p* = 0.727; Adult Control, *p* = 0.037; Adult EtOH, *p* = 0.118). *, *p* < 0.05; **, *p* < 0.01. The n number is provided within each bar

Y Maze spontaneous alternation is a widely-used behavioral test for measuring the willingness of rodents to explore new environments, to reflect spatial working memory. Rodents typically prefer to investigate a new arm of the maze rather than returning to one that was previously visited. To determine the potential effect of ABET on working memory, we performed the Y-maze test on control and ABET mice to examine their spontaneous alternation. The total number of arm entries and the percentage of triads of arm entries in which the mouse sequentially visited each possible arm without repeating were recorded (Fig. 3a). Our analysis did not find any significant differences in the total number of arm entries, suggesting that ABET did not have any effects of gross motor behavior in this assay (Fig. 3b, c). For spontaneous alternation, we adopted the quantification that we previously reported [10]. Both adolescent control and adult control mice had a percentage of alternation significantly above chance level (> 50% – one-sample t-test: adolescent control, *p* = 0.004; adult control, *p* = 0.037), while this was not the case for ABET adolescent and adult mice (Fig. 3d). These findings reveal that the effect of ABET on Y maze spontaneous alternation also persists into early adulthood.

**Fig. 4:**
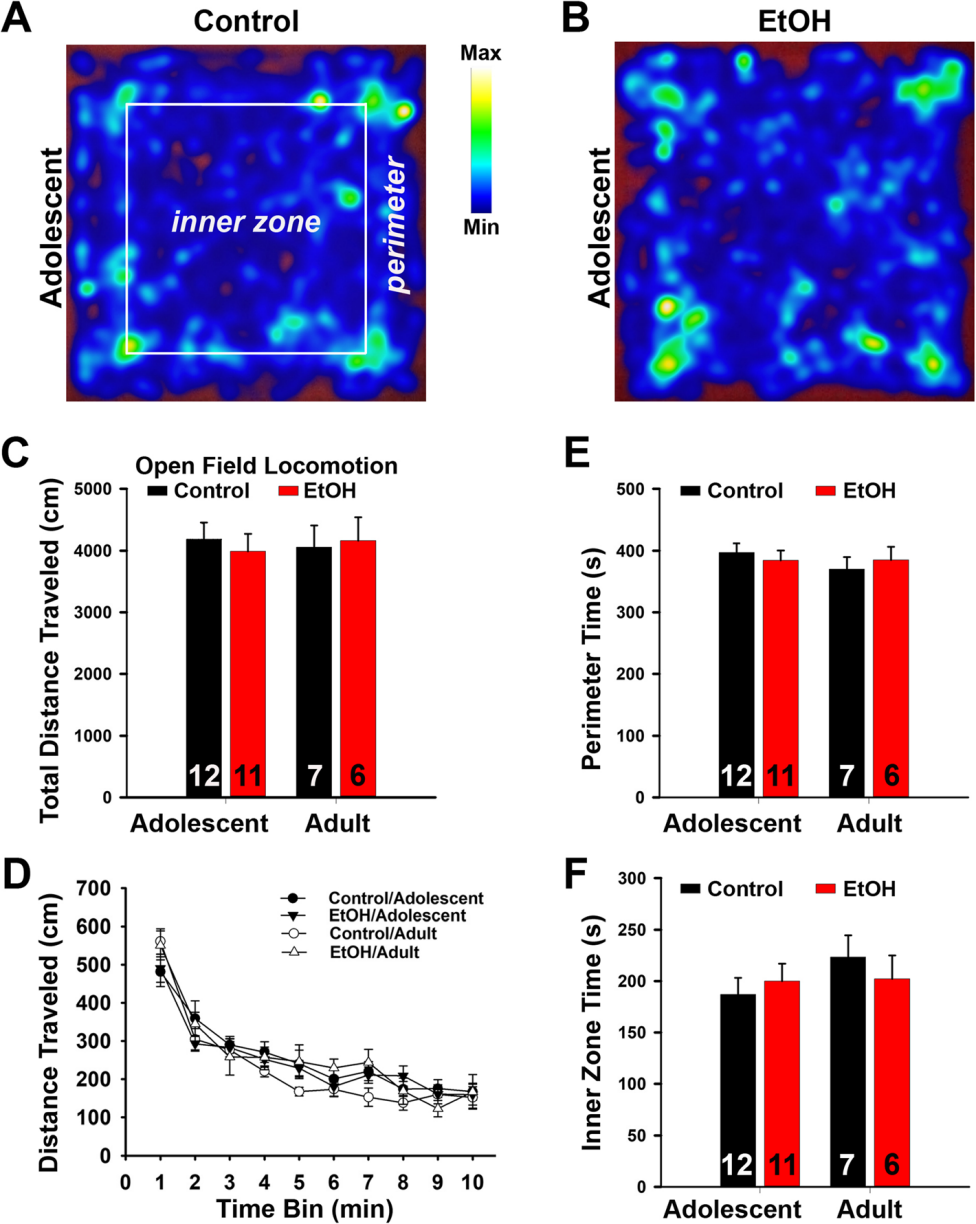
Overall locomotion was not affected by ABET in the open-field test. **a** Top view of the activity of a control adolescent mouse in a heat map. **b** Top view of the activity of an adolescent mouse with ABET. **c** Summary of total distance traveled. **d** Summary of the total distance traveled per minute. **e** Summary of time spent on the perimeter. **f** Summary of time spent in the center of the open-field. Two-way ANOVAs for total distance traveled (**c**), perimeter time (**e**), and inner zone time (**f**) did not reveal any significant effects for condition [total distance: F_1,32_ = 0.02, *p* = 0.89; perimeter time: F_1,32_ = 0.004, *p* = 0.95; inner zone time: F_1,32_ = 0.05, *p* = 0.83], age [total distance: F_1,32_ = 0.004, *p* = 0.95; perimeter time: F_1,32_ = 0.54, *p* = 0.47; inner zone time: F_1,32_ = 0.97, *p* = 0.33], or significant condition X age interactions [total distance; F_1,32_ = 0.22, *p* = 0.65; perimeter time: F_1,32_ = 0.58, *p* = 0.45; inner zone time: F_1,32_ = 0.78, *p* = 0.38]. The n number is provided within each bar

The open-field test is utilized to assess the overall locomotion and decreased time in the center of the arena may also reflect increased anxiety-like behavior. In this test, we observed no significant difference in total distance traveled between the ABET or saline groups (Fig. 4a–d). To more carefully examine locomotion in the open-field test, distance traveled was broken down into 1-min time bins (Fig. 4d). Repeated measures ANOVA revealed a significant effect of time, with all mice demonstrating reduced locomotion across the 10 min trial [F_9,297_ = 74.54, *p* < 0.001, two-way ANOVA] (Fig. 4d). However, the main effects of age and condition, as well as all interactions, were not significant (all *p’s* > 0.16). These results suggest that locomotor behavior was not impacted by ABET. We also found no effect of ABET on time spent in the perimeter or center of the open field for adolescents or adults (all *p*’*s* > 0.33). Taken together, these findings reveal that ABET has no effect on locomotor activity in three distinct assays, and it does not change the center/parameter time ratio in the open field test, which differs from the EPM results. This will be discussed later.

### ABET induced long-lasting reduction of PV+ neurons and MBP+ myelin density in the hippocampus

**Fig. 5:**
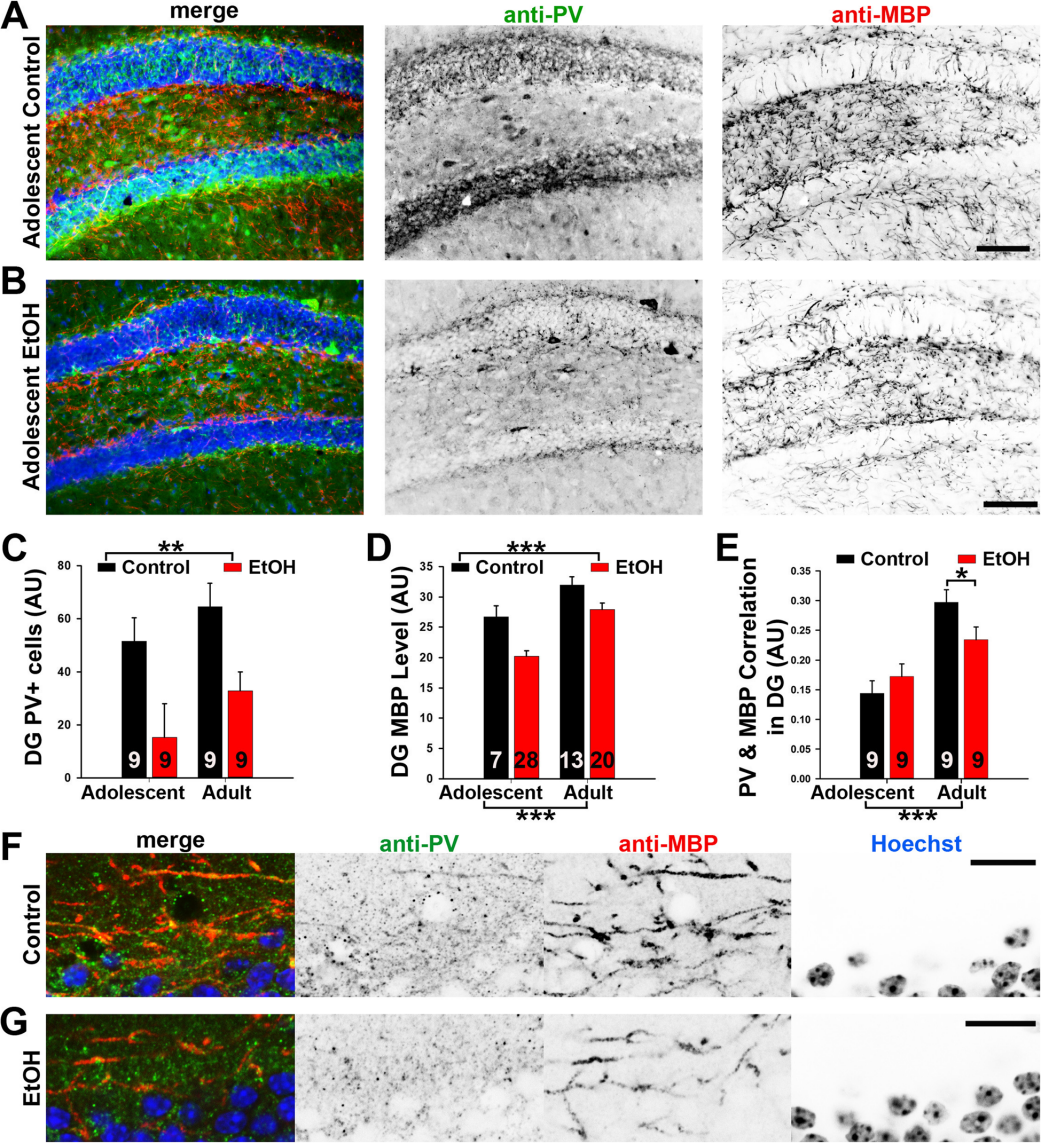
PV+ neurons and MBP+ myelin density in the hippocampus significantly reduced after ABET, which persisted into adulthood. The mice were cardiac perfused and their brains were fixed for anatomical studies after behavioral testing as described in Fig. 1. Representative images for PV (green in merge), MBP (red in merge) and Hoechst (blue in merge) co-staining in the dentate gyrus (Bregma − 2.06) of the control (**a**) and EtOH (**b**) adolescent mice. Signals are inverted in gray scale images. **c** Summary of PV+ cell body density in the hippocampus. **d** Summary of MBP+ myelin density in the hippocampus. Two-way ANOVA: **, *p* < 0.01; ***, *p* < 0.001. **e** Summary of PV and MBP correlation. Two-way ANOVA followed by post hoc Tukey’s test: *** *p* < 0.001; *, *p* < 0.05. **f** Confocal image for PV, MBP and Hoechst staining in control adolescent mouse. **g** Confocal image for PV, MBP and Hoechst staining in adolescent mouse with ABET. Scale bars, 250 μm in (**a**) and (**b**), 50 μm in (**f**) and (**g**)

To determine the potential impact of ABET on GM myelin, we performed anatomical studies focusing on the hippocampus and mPFC, two brain regions that play an integral role in anxiety and spatial working memory. The two regions are among the most sensitive targets of EtOH. More importantly, among multiple brain regions we scanned, these two regions displayed the biggest effects in response to ABET. Recent studies have shown that a significant portion of GM myelin within these two brain regions is formed on the axons of PV+ GABAergic interneurons [29, 42]. After behavioral testing, mice were cardiac perfused, and their brains were fixed, sectioned and co-immunostained for PV and myelin basic protein (MBP, a reliable mature myelin marker). While PV+ neuronal cell bodies were scattered in the hippocampus, PV+ axons were also observed throughout this region, with relatively higher intensity near the cell body layers of granule cells in the dentate gyrus (DG) (Fig. 5a). This pattern is consistent with the notion that PV+ GABAergic interneurons send their axons to form synapses around the soma and axon initial segments of their target neurons. Within the trisynaptic neural circuit of the hippocampal formation, there is no highly bundled myelinated axonal tracts giving rise to WM appearance. The hippocampus is a typical GM region in the CNS with the presence of myelin segments forming a mesh-like pattern (Fig. 5a). Since the DG region of the hippocampus has been linked to anxiety-like behaviors [3], we focused our anatomical analysis on this region.

ABET caused a marked reduction of PV+ cell density and MBP+ myelin segment density in the hippocampus of both adolescent and adult mice (Fig. 5a–d). Two-way ANOVA revealed a significant main effect for condition [F_1,20_ = 12.52, *p* = 0.002], with EtOH exposed (or ABET) mice having reduced PV+ cell body counts across both adolescence and adulthood (Fig. 5c). There were no significant effects of age [F_1,20_ = 2.52, *p* = 0.13, two-way ANOVA] and no condition X age interaction [F_1,20_ = 0.54, *p* = 0.82, two-way ANOVA] (Fig. 5c). Thus, there was no significant increase of the PV+ cell number between the adolescent and adult mice under our experimental condition. For MBP+ myelin segments, two-way ANOVA revealed that myelin density was significantly reduced in EtOH-exposed mice across both age groups [main effect of condition; F_1,64_ = 15.54, *p* < 0.001, two-way ANOVA], and importantly that myelin density was increased in adults regardless of EtOH exposure [main effect of age; F_1,64_ = 23.75, p < 0.001, two-way ANOVA]. The condition X age interaction was not significant [F_1,64_ = 0.81, *p* = 0.37, two-way ANOVA] (Fig. 5d). Therefore, in contrast to PV+ cell body density, the density of myelin segments in the hippocampus significantly increased between adolescence and adulthood.

To estimate how myelin segments of PV+ and PV- axons were impacted by ABET, we performed a correlation of PV and MBP co-localization (Fig. 5e). Pixel-by-pixel intensity was correlated between corresponding PV/MBP images. The average Pearson’s r value was calculated and subjected to a two-way ANOVA (Fig. 5e). This analysis revealed a significant condition X age of testing interaction [F_1,32_ = 4.57, *p* = 0.04, two-way ANOVA]. Post hoc pairwise analyses using Tukey’s test found higher PV/MBP correlations in adult control mice compared to adult ABET mice (*p* = 0.046), but not between the adolescent control and ABET mice (*p* = 0.35). Furthermore, adult mice demonstrated increased correlation values compared to adolescents for both control (*p* < 0.001) and ABET (*p* < 0.05) mice. Since both PV and MBP density were reduced and their correlation did not increase for both adolescent and adult mice after ABET (Fig. 5c–e), a majority of the observed reduction in myelin likely occurred in PV+ axons. This notion was confirmed by confocal microscopy, showing abundant PV+/MBP+ axons in the control (Fig. 5f) and reduced double-positive axons after ABET (Fig. 5g) in adolescent mice. Our results also indicate that there is a higher proportion of myelination on PV+ axons in adult mice compared to adolescent mice, but this increase is blunted by ABET.

### In the mPFC ABET caused persistent reduction of PV+ neurons and transient myelin reduction

**Fig. 6:**
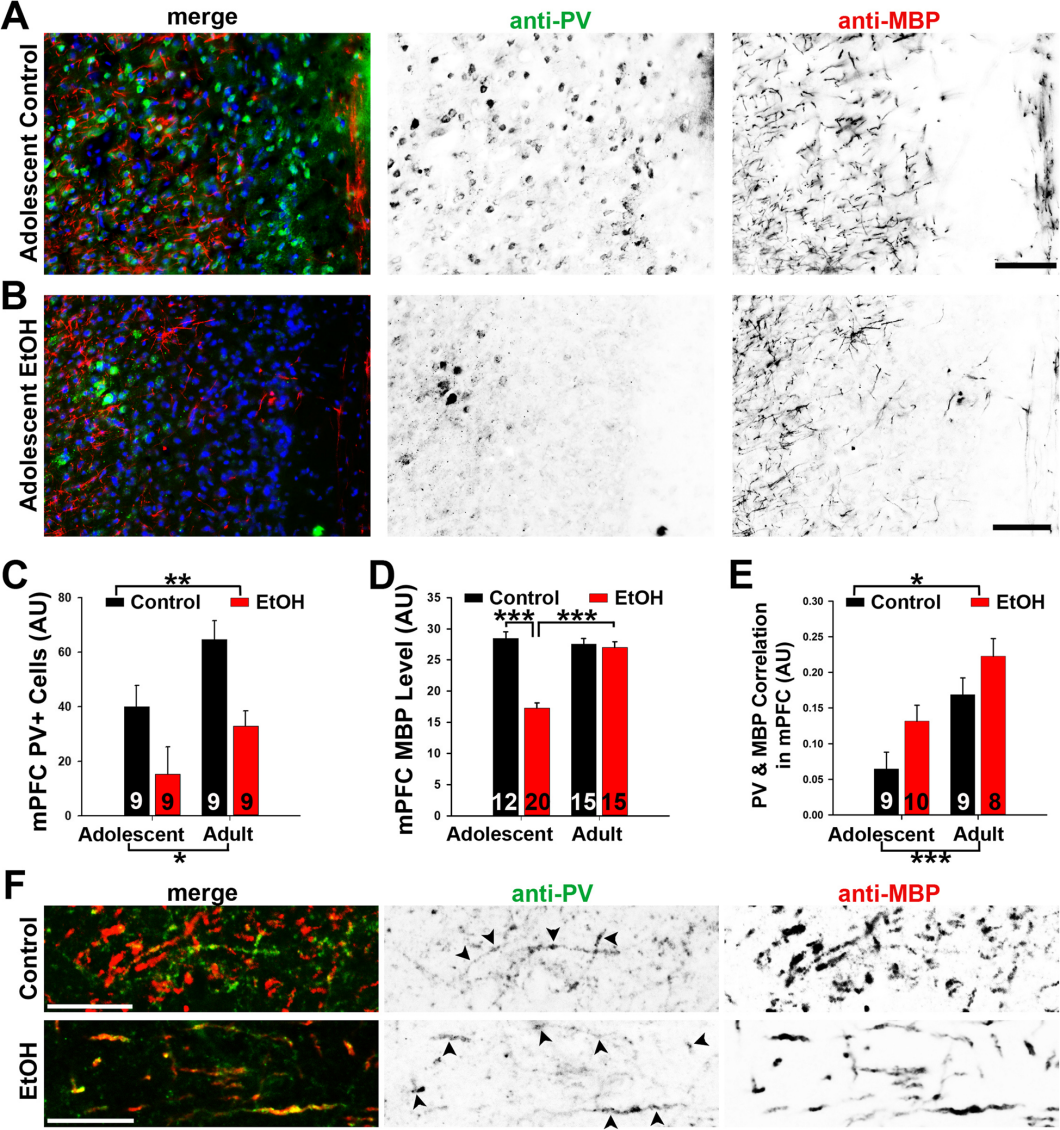
In the mPFC ABET caused a long-lasting reduction of PV+ neurons and transient myelin reduction. Representative images for PV (green in merge), MBP (red in merge) and Hoechst (blue in merge) co-staining in the mPFC (Bregma 1.98) of the control (**a**) and EtOH (**b**) adolescent mice. Signals are inverted in gray scale images. **c** Summary of PV+ cell body density in the mPFC. **d** Summary of MBP+ myelin density in the mPFC. **e** Summary of PV and MBP correlation in the mPFC. Two-way ANOVA followed by post hoc Tukey’s test: ***, *p* < 0.001; **, *p* < 0.01; *, *p* < 0.05. **f** Confocal images for PV and MBP staining in the mPFC of control (upper) and ABET (EtOH, lower) adolescent mouse. Arrowheads, PV+ axons colocalizing with MBP staining. Scale bars, 300 μm in (**a**) and (**b)**, 50 μm in (**f**)

We conducted a similar analysis of the effects of ABET on PV and MBP in the mPFC. PV+ neuronal cell bodies were observed across different layers of the mPFC, especially layers II and III, whereas MBP+ myelin segments formed a network-like pattern across the layers in adolescent mice (Fig. 6a). After ABET, both PV+ cell density and myelin density were significantly reduced in the mPFC of adolescent mice (Fig. 6a–d). For the PV+ cell density, two-way ANOVA revealed significant main effects for condition [F_1,19_ = 13.54, *p* = 0.002] and age [F_1,19_ = 7.49, *p* = 0.013], while the condition X age interaction was not significant [F_1,19_ = 0.21, *p* = 0.65] (Fig. 6c). Similar to the results in the hippocampus, ABET caused a reduction in PV+ cells that persisted into early adulthood. For MBP+ myelin density in the mPFC, two-way ANOVA revealed significant effects for condition [F_1,58_ = 39.05, *p* < 0.001], age [F_1,58_ = 22.17, *p* < 0.001], and condition X age [F_1,58_ = 32.57, *p* < 0.001] (Fig. 6d). Analysis of the significant interaction using Tukey’s post hoc test found that among control mice, age did not affect mPFC myelin density (*p* = 0.51), while adolescent ABET mice had lower myelin density than adults (*p* < 0.001) (Fig. 6d). Thus, in the mPFC, EtOH-exposed mice had significantly reduced myelin density than control mice immediately following ABET (*p* < 0.001), but this effect did not appear to persist into adulthood (*p* = 0.70) (Fig. 6d).

To determine how myelin segments of PV+ and PV- axons in the mPFC were impacted by ABET, we performed the correlational analysis of PV and MBP co-localization (Fig. 6e). Correlations were carried out for overlaid sets of PV/MBP images. Two-way ANOVA demonstrated significant main effects for condition [F_1,32_ = 6.53, *p* = 0.016] and age [F_1,32_ = 17.04, *p* < 0.001], while the condition X age interaction failed to reach significance [F_1,32_ = 0.07, *p* = 0.79] (Fig. 6e). These results were further confirmed by confocal microscopy. PV+/MBP+ axons were present in the mPFC of control adolescent mice and did not decrease after ABET, although ABET reduced myelin density (Fig. 6f). Taken together, our results suggest that, in sharp contrast to the hippocampus, demyelination mainly occurred for PV- axons in the mPFC immediately after ABET, while the proportion of myelin on PV+ axons significantly increased in early adulthood.

### Myelin damage was responsible for myelin density reduction right after ABET

**Fig. 7:**
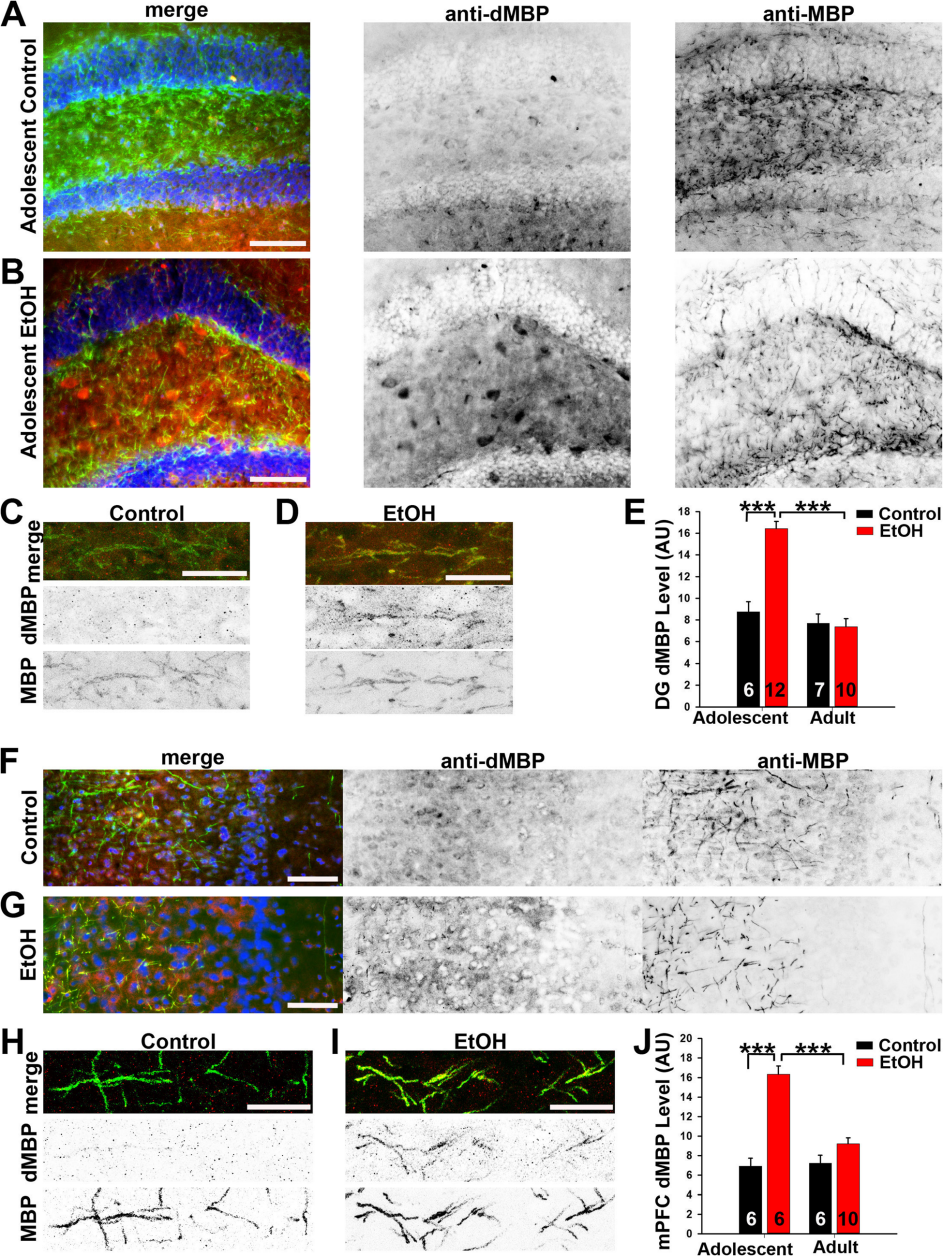
ABET markedly increased the dMBP level in adolescent but not adult mice. Representative images for dMBP (red in merge), MBP (green in merge) and Hoechst (blue in merge) co-staining in the dentate gyrus (Bregma − 2.06) of the control (**a**) and EtOH (**b**) adolescent mice. Signals are inverted in gray scale images. **c** Confocal image of control mouse. **d** Confocal image of EtOH (ABET) mouse. **e** Summary of dMBP density in the hippocampus. Representative images for dMBP (red in merge), MBP (green in merge) and Hoechst (blue in merge) co-staining in the mPFC (Bregma 1.98) of the control (**f**) and EtOH (**g**) adolescent mice. **h** Confocal image of control mouse. **i** Confocal image of EtOH (ABET) mouse. **j** Summary of dMBP density in the mPFC. Two-way ANOVA followed by post hoc Tukey’s test: *** *p* < 0.001. Scale bars, 250 μm in (**a**, **b**, **f**) and **G**; 25 μm in (**c**, **d**, **h**) and (**i**)

To determine whether the reduction in myelin resulted from demyelination or inhibition of myelination, we performed co-immunostaining using the anti-MBP antibody and an antibody against degraded MBP (dMBP) that was used to assess damage to myelin (Fig. 7). In the hippocampus, the dMBP level significantly increased in adolescent mice immediately after ABET, but it returned to normal in early adulthood (Fig. 7a–e). Two-way ANOVA revealed a significant condition X age interaction [F_1,31_ = 25.02, *p* < 0.001]. Tukey’s post hoc analyses of the significant interaction found that dMBP expression was increased in adolescent EtOH mice compared to controls (p < 0.001), while no such effect was found in adult ABET and control mice (*p* = 0.78) (Fig. 7e). In addition to some large aggregates, the dMBP staining signals appeared to colocalize with some MBP+ myelin segments after ABET (Fig. 7a–d).

Similar results were found in the mPFC for dMBP expression (Fig. 6f–j). Two-way ANOVA indicated a significant condition x age interaction [F_1,24_ = 22.39, *p* < 0.001]. As in the DG, Tukey’s post hoc analyses of the interaction found increased dMBP expression in adolescent EtOH mice compared to controls (*p* < 0.001) while no significant difference in mPFC dMBP expression between adult control and adult EtOH mice (*p* = 0.07) (Fig. 7j). The dMBP staining signals also appeared to colocalize with some MBP+ myelin segments after ABET (Fig. 7f–i). Taken together, demyelination is most likely induced by ABET, and accounts for, perhaps not entirely, the reduction in myelin observed shortly after ABET, but it apparently does not persist into early adulthood.

## Discussion

Using the ABET mouse model, we found that this treatment induced a long-lasting increase in anxiety-like behavior, a persistent reduction in working memory, and differentially altered interneuron myelination in the hippocampus and mPFC. Although ABET reduced PV+ neurons and myelin density in both regions, our results show for the first time that there are regional differences in interneuron demyelination caused by this EtOH treatment. Taken together, our findings suggest that brain-region specific alterations of PV+ interneurons and their myelin may play a key role in the maladaptive physiological and behavioral changes induced by adolescent binge drinking.

Our EPM results indicate that after ABET, adolescent mice had significantly less percentage time in the open arms/(open + closed arms), an effect that endured into adulthood (Fig. 2). This result is consistent with prior findings showing that binge alcohol treatment persistently increased anxiety-like behavior using either an open-field test [9] or a light/dark box exploration test [37]. However, our open-field test (10 min total) failed to detect any significant change in center time caused by ABET (Fig. 4), which is different from the result of the open-field test in the earlier study (2 h total) [9]. This most likely resulted from differences in the durations of the tests being used in these studies. We used a shorter time (10 min) in our open-field assay to primarily examine locomotion (Fig. 4). Actually, our result is likely consistent with the initial period of the long open-field test [9]. Nonetheless, anxiety is a very complex behavior and it is not surprising to see differences between tests, which often reflect different types of anxiety [35]. Importantly, as observed in other studies [9], we did not observe any ABET-associated changes in locomotor activity (Figs. 3 and 4).

Our result using the Y-maze test shows that ABET significantly reduced spontaneous alternation in adolescent mice, suggesting impaired working memory, and this impairment did persist to some extent into early adulthood (Fig. 3). This seems consistent with a recent study, using the rewarded alternation test, that showed that EtOH binge-treated rats of both sexes displayed spatial working memory deficits [45]. Interestingly, another study using a rat model of alcohol binge drinking showed that heavier drinking predicted worse performance on the T-maze working memory task in adulthood [44]. In mice, adolescent intermittent EtOH did not impact adult Barnes Maze learning, but caused reversal learning deficits in adults [9]. On the other hand, another recent study showed that binge-treated rats were impaired during the first attention set shift on an operant set-shifting task, but displayed no difference in spatial exploration, learning and reversal, or novel object recognition [16]. In DBA/2 J adolescent mice, binge treatment caused long-term memory deficits using the novel object recognition test [48]. These differences most likely result from differences in the tests and animal strains used in these studies. Nonetheless, establishing the specific cognitive deficits caused by alcohol binge drinking likely still requires more extensive investigation.

Since GM myelination is a long process and coincides with periadolescent development, the present study mainly focused on the effects of ABET on GM myelin. Our results show that GM myelin in both the hippocampus and the mPFC was significantly reduced after ABET (Figs. 5 and 6). Notably, the reduced myelin levels appeared to persist into adulthood in the hippocampus, but not the mPFC (Figs. 5 and 6). This appears somewhat different from the conclusion of a recent paper that binge drinking in adolescent male rats reduced myelin density in the mPFC in adulthood [44]. However, it is important to note that in their alcohol dependence protocol, rats were subjected to EtOH vapor exposure before myelin staining in adulthood [44]. Consistent with our results (Fig. 6), right after binge drinking there was a clear reduction of prefrontal myelin [44]. Our results show that reduced myelin in adolescents by ABET returned to control levels in early adulthood, presumably through more robust remyelination, which may be an interesting topic for future instigation.

Recent studies showed that a significant proportion of GM myelination in the cortex and hippocampus of both rodents and humans is localized to the axons of local inhibitory GABAergic interneurons and that almost all myelinated GABAergic axons are from PV+ fast-spiking interneurons [29, 42]. Thus, we performed co-staining for PV and MBP to determine the effect of ABET on interneuron myelination. Interestingly, our results showed that ABET significantly reduced PV+ interneurons in both the hippocampus and the mPFC, and the reduction persisted into adulthood in both regions (Figs. 5 and 6). By using designer receptors exclusively activated by designer drugs (DREADD) technology, a recent study showed that proper control of PV+ interneurons activity in the dentate gyrus of the hippocampus is critical for regulation of the anxiety, social interaction and fear extinction [52]. This is consistent with our results showing persistently reduced PV+ neurons in the hippocampus and persistently heightened anxiety-like behavior following ABET (Figs. 2 and 5). However, it currently remains unclear whether ABET-induced reduction of PV+ cell density resulted from inhibited PV+ cell proliferation during adolescent development and/or PV+ cell death. Moreover, we also cannot exclude the possibility that ABET specifically reduces PV expression without affecting the functions of FS GABAergic interneurons. These possibilities remain to be investigated in future studies.

To the best of our knowledge, the present study is the first report that ABET induces myelin alterations in a region-specific manner. Our results of PV/MBP correlations suggest that, in the hippocampus, ABET reduced myelin primarily on PV+ axons, and this effect persisted into adulthood, whereas PV+ axonal myelination markedly increased in adult control mice (Fig. 5e). In the mPFC, ABET apparently reduced myelin mainly on PV- axons (presumably from excitatory projection neurons), and this effect persisted into adulthood (Fig. 6e). Since overall mPFC myelin recovered while PV+/MBP+ axons increased at the adult time point in ABET mice, PV−/MBP+ axons likely remained low compared to control subjects. Importantly, we found that levels of myelinated PV+ axons are much higher in the hippocampus than that in the mPFC (Figs. 5e and 6e). This varied degree of myelination for PV+ interneurons in different brain regions is actually consistent with the findings of another recent study [42]. Reduced level of myelin, especially at the adult stage, can result from demyelination and/or inhibition of myelination. Our results using dMBP staining clearly showed that ABET-induced acute myelin damage in both the hippocampus and the mPFC (Fig. 7). The dMBP signals returned to normal levels in adult mice, suggesting that differences in the levels of myelin at the adult stage involve altered myelination and/or remyelination processes.

These changes in PV+ interneurons and myelin may have profound effects on the computation of neural networks. For instance, in the hippocampus ABET causes a reduction in PV+ interneurons together with their myelin, which may lead to persistently reduced inhibitory capacity of the neural network. On the other hand, in the mPFC, ABET caused a significant reduction of PV+ interneurons but demyelination was primarily restricted to PV- projection neurons, which may lead to reduced inhibition and synchronization of the neural network. Importantly, reduced density of PV+ interneurons in the PFC and/or hippocampus is associated with cognitive deficits in rodent models [7, 26], and human patients with schizophrenia [24]. Reduced prefrontal and hippocampal myelination during postnatal development may lead to abnormal social interaction, anxiety and working memory [27, 50]. These appear consistent with our findings here. Nonetheless, the exact physiological and behavioral impact of these brain-region specific effects of ABET on interneuron myelination in the hippocampus and mPFC currently remain to be fully elucidated. Of note, adolescent alcohol exposure can persistently impact adult neurobiology, in which different neurotransmission systems have been implicated [12]. Extensive future studies will be needed to fully understand the role of interneuron demyelination of different brain regions (e.g. other regions of cerebral cortex and limbic system, etc) in the pathogenesis associated with adolescent binge drinking. Moreover, it is important to note that the present study has used male mice in the ABET model. Potential gender difference in adolescent binge drinking will be an interesting topic for future investigation.

## Conclusion

Taken together, our findings suggest that differential alterations of myelination of PV+ and PV- axons in the hippocampus and mPFC may play a key role in the neuroadaptive changes induced by adolescent binge drinking, and may represent a novel target for the development of new treatment strategies for AUDs
